# Accessing Mental Health Services in Africa: Current state, efforts, challenges and recommendation

**DOI:** 10.1016/j.amsu.2022.104421

**Published:** 2022-08-18

**Authors:** Aderinto Nicholas, Opanike Joshua, Oladipo Elizabeth

**Affiliations:** Department of Medicine and Surgery, Ladoke Akintola University of Technology, Ogbomoso, Nigeria; Department of Medical Laboratory Science, Federal Neuropsychiatric Hospital, Yaba, Lagos, Nigeria

**Keywords:** Mental health, Africa, Health services

## Abstract

The COVID-19 pandemic brought to the surface the dwindling state of mental health services in Africa. While most countries have policies targeted at mental health problems, these policies are often weak and outdated to combat the present challenges. Africa faces various challenges in mental health delivery, ranging from inadequate staffing to sociocultural stigma and lack of effort from the government in terms of policies and funding. Many countries do not have the budgetary allocation for mental health. while those with budgetary allocation spend less than 5% of government health expenditure on mental health. Considering the continent's socioeconomic difficulties, it is almost a given that mental health illnesses will be on the rise. The continent's growing population, which is majorly youth, means that mental health services will be in high demand in the coming years. Therefore, the relevant stakeholders must look into the challenges and respond with a sense of urgency.

## 1 Introduction

Mental health services in Africa have suffered from many challenges. The situation has not shown any sign of change in recent years, especially in light of the COVID-19 pandemic. Lack of resources and commitment from public stakeholders continue to hinder the development of mental health care in most African nations, where specialized psychiatric care is primarily provided in urban situated mental institutions. Even though many African nations have policies to address mental health problems, these policies are often weak and outdated to combat the present challenges. It, therefore, becomes challenging to pinpoint areas of need, organize the efforts, and decide on the course of legislation.

In essence, issues related to mental health continue to be neglected across Africa. For instance, a poll on mental health conducted by the Africa Polling Institute (API) and EpiAFRIC in Nigeria, the most populous nation in Africa, reveals a poor awareness of mental health, with the majority of respondents unaware that they have mental health illnesses [1]. Most patients with mental health conditions often seek spiritual interventions because mental health illnesses are frequently linked to cultural and ancestral roots. Government support for mental health initiatives continues to be pitifully inadequate. Failure to collaborate to address this health crisis has enormous adverse effects on human potential and unnecessary suffering for the African population.

The Current Situation of Mental Health Services In Africa.

The current look of Africa's mental health is gloomy, and despite tries, the situation has not changed. Africa has 1.4 mental health workers per 100 000 people, a low figure beside the global average of 9 workers per 100 000 people [2]. Also, there has been a very small number of practising psychiatrists. Additionally, the global annual rate of visits to outpatient mental health facilities is 1051 per 100 000 people, but when Africa is zoomed in, it is only about 14 per 100 000 [3]. In Sierra Leone, it is estimated that 98% of people lack access to mental health care. In addition, there are not many studies on psychiatric diseases in Africa [4]. According to a study, only 3% of clinical trials for mental health were carried out in low- and middle-income nations, with African nations making up the bulk of this faction [5].

The demand for mental health treatments in Africa has increased recently. Africa's population increased by 49% over the previous two decades [6]. The number of years lost to disability due to mental and substance use disorders also increased concurrently by 52% [7]. As a result of mental health issues, 17.9 million years were lost to disability in 2015 [8].

Over the next 30 years, Africa's population, primarily comprised of young people, will nearly double [9]. Considering the continent's socioeconomic difficulties, it is almost a given that mental health illnesses will be on the rise. Africa hence requires robust mental health care on all fronts. The scientific community and other relevant public parties must intensify their efforts to prepare for this impending calamity.

## 2 Challenges

While there are peculiar barriers which have roots in the sociocultural dynamics of the population, barriers to mental health services in Africa are similar to barriers affecting general healthcare delivery on the continent, with some peculiarities.

Generally, 96.3% of these barriers are attitudinal. There are also reports of structural barriers such as financial and care availability, including treatment facilities and staff whose distribution is influenced by sociodemographic factors [10].

There remains a dearth in the number of mental health facilities in Africa, and the available facilities are inadequate for population needs. For example, Egypt has 15 mental hospitals across the country, and the inpatient units collectively hold less than ten beds per 100 000 population. Nigeria has seven mental hospitals owned by the government, which do not hold more than four inpatient beds per 100 000 population [11]. In Uganda, there was one national mental hospital and 1.64 beds per 100 000 of the population in inpatient units [12]. There are few or no specialized centres for children and adolescents. Most of the centres are located in urban areas, restricting access to more than 50% of the population living in rural areas, which usually comprise 65% of patients [13]. The absence of a defined catchment basis for siting service delivery also confounds the problem and invariably leads to patients losing to follow-up, especially when they travel long distances [14].

In most African countries, the number of available Psychiatrists is primarily less than the recommended 1 to 10 000 population ratio [15]. Nurses, auxiliary staff, and medical and health assistants form most of the insufficient health staff. Furthermore, integration in general health care is low. Practitioners in general settings are also potentially incapacitated to provide essential services. The paucity of staff for most patients meant longer waiting times with consequent adverse effects on work.

Few countries, such as Libya, lack a standalone mental health policy, but the majority with a policy did not deploy financial and human resources to implement or monitor progress [16]. The absence of a policy or a non-functional policy makes it impossible to reach clients with needs for mental health services leading to ineffective service delivery and potential wastage of resources.

In general, payment for healthcare is mainly out-of-pocket, putting a significant burden on individuals and families and sometimes causing severe financial strains. This generally leads to poor treatment outcomes consequent upon the inability to procure medications. Many countries do not have the budgetary allocation for mental health. while those with budgetary allocation spend less than 5% of government health expenditure on mental health.

The sociocultural bias toward mental health disorders remains an age-long barrier to seeking care and has been a global problem. Mental health patients are considered dangerous, and 82% of the population would decline in their fundamental social interactions. These assertions are uniformly distributed among the community [17]. Patients are afraid of being stigmatized and choose not to seek help, a probable indication of a lack of trust in the confidentiality of the healthcare system. Sometimes, family members decide not to seek care for the same reason. Also, help is sometimes sought from traditional and spiritual healers as mental health is sometimes believed to be a spiritual problem [18].

Personal perspectives of the condition discourage patients from seeking help as patients with mild disorders are generally less likely to seek help, and half want to handle the condition independently. 17% of patients do not think the problem was severe and assumed it would go away on its own, a pointer to a generally low level of knowledge and attitude to mental health.

## 3 Recommendations

Integrating mental health services into primary health care is essential to improving accessibility and eliminating many structural barriers [18]. This should include capacity building of health workers, especially other medical non-doctor staff who comprise the more significant percentage of personnel in PHCs to provide prescription and referral services using tools like the mhGAP. And likewise availability of psychotropic drugs, especially for chronic conditions. The capacity of community-based mental health services and tertiary centres should be boosted to accommodate more patients by increasing personnel, infrastructure and general funding.

Stigma from misconceptions on mental health has no preferential sociodemographic distribution; hence community-based advocacy and enlightenment programs should target all population groups, albeit with specific themes. While there is an increase in several non-governmental advocacy groups, activities must be periodically accessed to leverage successes and improve outcomes.

There is a need to formulate policies in countries where there are none and strengthen them where they are present. These policies must outline strategic visions, action plans, and assessment tools and be backed by a legal framework. The need to decentralize care must be recognized with the specific deployment of human and financial resources to identified catchment areas [19]. Inter-sectorial collaboration and community participation should be enshrined in the document. Likewise, policy should be periodically monitored and implementation adequately accessed.

The most significant barrier to effective mental health policy is inadequate funding. Adequate funding is essential for medications, protocols, staffing and infrastructure. It is essential to increase the allotted quota for mental health provided by budgetary allocations. Spending hitherto concentrated in tertiary care should be decentralized to include lower care strata. Insurance schemes should be improved to capture more users. With many governments facing economic difficulties, public-private partnership is essential to sustain an accessible mental health system on the continent.

## 4 Conclusion

It is evident that without necessary actions from every stakeholder involved in delivering mental health services in Africa, catastrophe is only a matter of time. The young African population, with the absence of an adequate number of mental health service providers and robust policies, means mental health illnesses might become a health emergency. However, there is still a window of opportunity to reimagine the mental health services in the continents. Relevant stakeholders from different countries must come together to develop a long term for the continent.

## Ethical approval

Not Applicable.

## Sources of funding

None.

## Author contributions

Aderinto Nicholas: Conceptualization, Project administration, Writing-review and Designing.

Aderinto Nicholas: Collection and assembly of data.

Aderinto Nicholas: Reviewed and edited the final draft.

Manuscript writing: All authors.

Final approval of manuscript: All authors.

## Registration of research studies

1. Name of the registry: Not Applicable
2. Unique Identifying number or registration ID: Not Applicable
3. Hyperlink to your specific registration (must be publicly accessible and will be checked): Not Applicable

## Guarantor

Aderinto Nicholas: Principal Investigator(PI).

## Consent

Not Applicable.

## Data availability statement

Not Applicable.

## Declaration of competing interest

No conflicts of interest declared.
